# Single‐cell transcriptional consequences of leukaemogenic *SETBP1* mutations

**DOI:** 10.1111/bjh.70452

**Published:** 2026-03-25

**Authors:** Mi K. Trinh, Nathaniel D. Anderson, Matthew D. Young, Holly J. Whitfield, Conor Parks, Toochi Ogbonnah, Agnes Oszlanczi, Di Zhou, Emilia R. Robertson, Angus Hodder, Rebecca Thomas, Karin Straathof, Stuart Adams, Jack Bartram, Nuala Summerfield, Sam Behjati

**Affiliations:** ^1^ Wellcome Sanger Institute Hinxton UK; ^2^ Great Ormond Street Hospital for Children NHS Foundation Trust London UK; ^3^ UCL Cancer Institute London UK; ^4^ Great Ormond Street Biomedical Research Centre London UK; ^5^ UCL Great Ormond Street Institute of Child Health London UK; ^6^ The Schinzel–Giedion Syndrome Foundation Chichester UK; ^7^ Department of Paediatrics University of Cambridge Cambridge UK; ^8^ Cambridge University Hospitals NHS Foundation Trust Cambridge UK

**Keywords:** myelodysplastic leukaemia, Schinzel–Giedion syndrome, *SETBP1* mutation, single‐cell transcriptional dysregulation

To the Editor,

Cellular effects of oncogenic mutations are traditionally studied in model systems, such as cell lines or animal models. While these approaches afford precise control over confounders and enable experimental manipulation, their inherent weakness is the non‐native habitat in which mutations are placed. Natural genetic variation, by contrast, enables the investigation of mutations within their pathophysiological context. Following advances in single‐cell mRNA sequencing (scRNA‐seq) technologies, which provide unbiased readouts of cellular transcriptional effects of mutations, it has become tractable to study consequences of individual oncogenic mutations from natural variation. A major limitation to this approach is the availability of adequately stored human tissues, especially in the context of rare mutations and disorders.

Schinzel–Giedion syndrome (SGS) is among the rarest human developmental disorders.^1^, ^2^ At the time of writing, two children with SGS were known to be alive in the United Kingdom. Worldwide, 40 individuals are known to be alive (personal communication by The Schinzel–Giedion Syndrome Foundation [https://sgsfoundation.org]). The condition is caused by germline activating missense mutations in the SKI domain (hotspot region) of the *SETBP1* gene.^3^ *SETBP1* encodes for SET binding protein 1, a widely expressed transcription regulator that exerts its function via control of epigenetic factors.^4^ These hotspot mutations result in prolonged stabilisation of SET protein, which inhibits the protein phosphatase 2A (PP2A). Most affected individuals are not born alive or do not survive infancy. A small number of children live beyond infancy, although the syndrome is ultimately life‐limiting through complex effects on many organ systems.^1^, ^2^ SGS predisposes to cancer.^5^ The same hotspot mutations that underpin SGS are the principal cancer‐causing (‘driver’) variants in atypical chronic myeloid leukaemia (aCML) of adults.^6^ In leukaemia of children, *SETBP1* is infrequently mutated, being largely confined to rare cases of juvenile myelomonocytic leukaemia (JMML)^7^ and myelodysplastic syndrome (MDS; a pre‐leukaemic neoplasm).^8^, ^9^

In an effort to delineate the effects of earliest leukaemogenic *SETBP1* mutations in quantitative molecular terms, we studied normal peripheral blood cells of children with SGS by scRNA‐seq (Figure 1A; Table S1). In partnership with The Schinzel–Giedion Syndrome Foundation (https://sgsfoundation.org/), we obtained fresh or viably frozen peripheral blood cells from five individuals directly or from the CombinedBrain tissue bank (https://combinedbrain.org/biorepository), with institutional and, where required by national law, external ethical approval in place. We subjected blood cells to scRNA‐seq through the 10× Chromium platform using standard protocols. In addition, we performed whole genome DNA sequencing to a coverage of at least 30× of blood‐derived DNA to determine the precise *SETBP1* mutation in each individual (Figure S1a). To contextualise these data, we generated additional single‐cell transcriptomes from a healthy donor and incorporated the published scRNA‐seq data from peripheral blood of healthy children^10^ to serve as normal reference. We also obtained publicly available scRNA‐seq data of two paediatric myeloid neoplasms—one harbouring a *SETBP1* hotspot mutation and one driven by a *GATA1* mutation (i.e. lacking *SETBP1* alterations)^11^ (Figure 1A). In addition, we re‐analysed published bulk mRNA‐seq datasets of *SETBP1*‐mutant and wild‐type aCML^6^ and JMML.^12^ Our overall analytical strategy was to derive somatic and germline *SETBP1* gene ‘modules’ (i.e. a list of genes altered in the presence of *SETBP1* mutation) and identify a shared myeloid‐focused transcriptional signature corresponding to the overlap between the somatic and germline modules for further analysis, given the well‐established association of *SETBP1* with myeloid malignancies.

**FIGURE 1:**
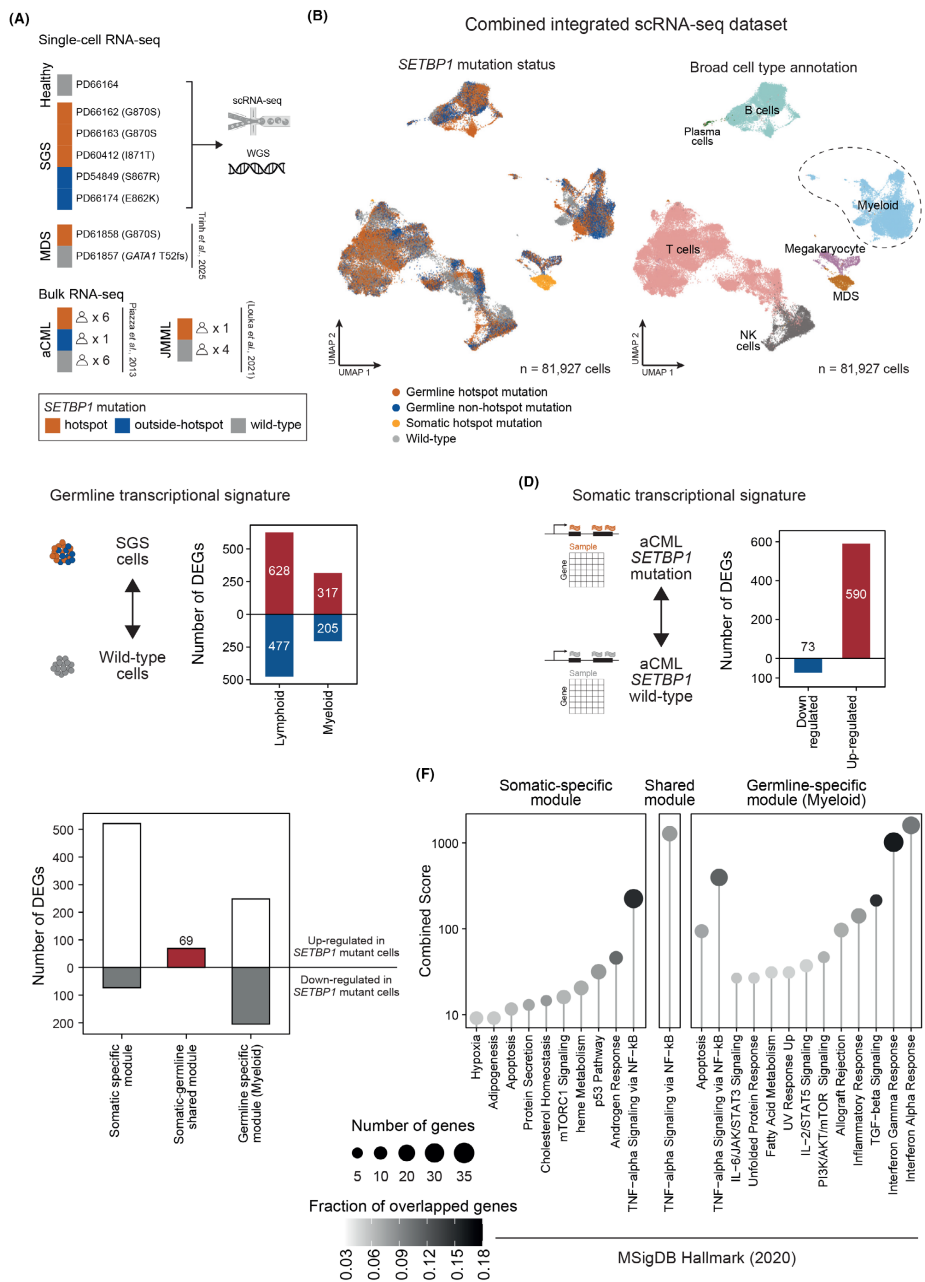
Distillation of the core transcriptional signature induced by *SETBP1* mutations. (A) Overview of datasets utilised in this study, including newly generated and publicly available single‐cell and bulk transcriptomic data. (B) Uniform Manifold Approximation and Projection (UMAP) visualisation (Harmony‐integrated) of single‐cell RNA‐seq data from peripheral blood samples of children with germline *SETBP1* mutations, one healthy donor and two children with myelodysplastic syndrome (MDS) with and without somatic *SETBP1* mutations. Cells (dots) are coloured by (left) *SETBP1* mutation status or (right) broad cell type categories. (C) Bar plot showing the number of markers significantly associated with *SETBP1*‐mutant cells compared to their counterparts from the healthy donor, across cell types of the lymphoid and myeloid lineages (see Supporting Information: Methods). Red indicates upregulated genes, and blue indicates downregulated genes in *SETBP1*‐mutant cells. The full gene module is detailed in Table S2. (D) Bar plot showing the number of differentially expressed genes (DEGs) between bulk atypical chronic myeloid leukaemia (aCML) samples with *SETBP1* hotspot mutations and those with wild‐type *SETBP1*. Red indicates upregulated genes, and blue indicates downregulated genes in *SETBP1*‐mutant aCML samples. The full gene module is detailed in Table S3. (E) Bar plot showing the number of unique and overlapping genes between the germline (myeloid) and somatic gene signatures as defined in (C, D) respectively. The red bar indicates genes upregulated in both modules. White bars represent genes upregulated only in one module, and grey bars indicate uniquely downregulated genes in *SETBP1*‐mutant samples. No downregulated genes were shared between the germline and somatic modules. (F) MsigDB Hallmark gene set enrichment analysis of the upregulated DEGs from each transcriptional module in panel (E). Only terms with significant enrichment (FDR < 0.1) are shown. Dot size corresponds to the number of differentially expressed genes present in each pathway, and colour indicates the fraction of the entire pathway overlapped with differentially expressed genes.

We subjected scRNA‐seq data (five children with SGS; two children with MDS; one healthy donor) to standard quality control procedures, including removal of ambient mRNAs and doublets, and cell‐type annotation. We thus recovered 81 927 high‐quality cells, of which 1753 were neoplastic cells derived from MDS cases (Figure 1B). Our scRNA‐seq dataset successfully captured the main haematopoietic cell types across both myeloid and lymphoid lineages from all samples (Figure 1B; Figure S1b,c). Previous work has suggested that *SETBP1* mutations skew haematopoiesis towards the myeloid lineage.^13^ Consistent with this, we observed a significantly higher proportion of myeloid cells and a reduction in NK/T cells in peripheral blood samples from individuals with SGS or atypical SGS compared to controls (Figure S1d). All paediatric samples were under 10 years of age and analysed as a single age group due to limited sample size; age‐stratified analyses in larger cohorts may reveal further differences. *SETBP1* expression among peripheral myeloid cells was confined to CD16+ monocytes and plasmacytoid dendritic cells in both SGS and healthy donors (Figure S2a). This may provide an explanation for the specificity of leukaemogenic *SETBP1* mutations to myeloid neoplasms of monocytic differentiation.

In a first set of analyses, we determined the transcriptional consequences (‘module’) of germline and somatic *SETBP1* mutations, as well as their overlap. First, we derived a germline *SETBP1* module by performing differential gene expression profiling, examining myeloid and lymphoid lineages separately, of SGS peripheral cells against those from the healthy donor (Figure 1C; Table S2). Similarly, we defined the somatic *SETBP1* module using the published bulk aCML dataset (from Piazza et al.^6^), comparing *SETBP1*‐mutant to wild‐type cases (Figure 1D; Table S3). This bulk dataset was chosen because it provides a well‐powered comparison across multiple *SETBP1*‐mutant and wild‐type samples within a single myeloid neoplasm. Our two MDS single‐cell datasets were subsequently used for independent validation of this module. We then intersected the somatic and the germline myeloid *SETBP1* module to identify a shared transcriptional signature (Figure 1E; Tables S2 and S3). These two modules converged on a core set of 69 genes consistently upregulated in both contexts, with no shared downregulated genes. The shared gene module was characterised thematically by ‘TNF alpha signalling’ as annotated with the MSigDB Hallmark database (Figure 1F). Of note, enrichment of ‘TNF alpha signalling’ was also observed in the germline lymphoid *SETBP1* module (Figure S2b), suggesting that *SETBP1* mutations may activate a lineage‐agnostic inflammatory transcriptional programme.

In a second set of analyses, we sought to validate these *SETBP1* modules in independent datasets. First, we examined single‐cell transcriptomes from a child with MDS harbouring a *SETBP1* hotspot mutation (PD61858, Figure 1B) and compared to those from a (non‐syndromic) child with *SETBP1*‐wild‐type MDS (PD61857, Figure 1B). It is important to note, in this context, that MDS cells are in the majority non‐transformed. Examining imprints of each module by scoring for its enrichment in individual cells in these data showed increased scores in *SETBP1*‐mutant MDS cells, with the highest score imparted by the shared module (Figure 2A). We then assessed the modules in the bulk JMML transcriptomes.^12^ Despite the inherent imprecision of bulk transcriptomes, there were clear imprints of each *SETBP1* module in JMML, with *SETBP1*‐mutant samples exhibiting higher signals than *SETBP1*‐wild‐type JMML (Figure 2B). There was a noteworthy difference in the enrichment of the germline‐specific *SETBP1* signal compared to both the somatic‐specific and shared signal. A possible explanation for the markedly lower germline *SETBP1* score in JMML might be that the germline‐specific signature was solely derived from non‐transformed cells. We extended this analysis to the BeatAML cohort,^14^ computing enrichment of the shared *SETBP1* module across samples. No clear distinction was observed between *SETBP1*‐mutant and wild‐type cases (Figure S3). This is likely driven by the heterogeneity of disease types and other co‐occurring driver mutations in these samples, which may induce overlapping transcriptional changes and obscure *SETBP1*‐specific signals.

**FIGURE 2:**
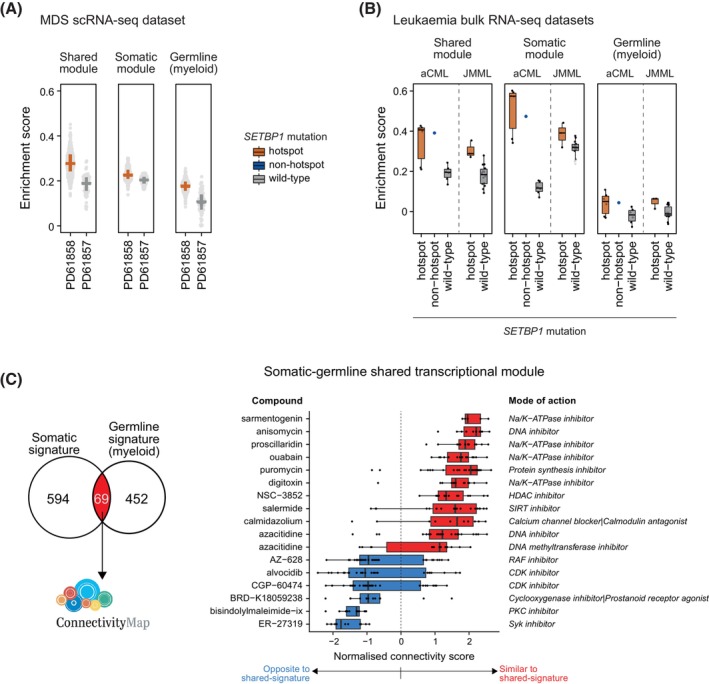
Imprints of the *SETBP1*‐mutation transcriptional modules in myeloid neoplasms. (A) Enrichment scores (*y*‐axis) of the three transcriptional modules associated with *SETBP1* mutations (as defined in Figure 1E) across individual neoplastic cells (grey dots) from the myelodysplastic syndrome (MDS) single‐cell mRNA sequencing (scRNA‐seq) dataset (*x*‐axis). Crossbars indicate the interquartile range of enrichment score distribution, coloured by *SETBP1* mutation status: Orange = hotspot mutation, blue = non‐hotspot mutation, grey = wild type. (B) Per‐sample enrichment score of the three transcriptional modules (*y*‐axis) in bulk RNA‐seq datasets of atypical chronic myeloid leukaemia (aCML) (from Piazza et al.^6^) and juvenile myelomonocytic leukaemia (JMML) (from Louka et al.^12^). Boxplots are coloured by *SETBP1* mutation status, as detailed in panel (A). (C) (Left) The shared *SETBP1* module was queried against the Connectivity Map database of perturbation‐induced transcriptional changes. (Right) Boxplot summarising normalised connectivity scores (*x*‐axis) comparing transcriptional perturbation signatures upon treatment with different compounds (*y*‐axis) in various cell lines (black dots) from the database against the shared *SETBP1* module. Only compounds with absolute median normalised connectivity scores above 1 are shown. Red indicates compounds with high similarity to the shared *SETBP1* module, whereas blue highlights compounds with strong opposing signatures.

Having derived a set of differentially expressed genes that encompass transcriptional effects of *SETBP1* mutation, we explored potential therapeutic strategies targeting these gene modules. Applying a previously described approach (see Supporting Information: Methods), we queried the Connectivity Map^15^—a compendium of drug‐induced transcriptional changes—to identify existing agents that may reverse the effects of the shared *SETBP1* module (Figure 2C; Figure S4). The query yielded a number of agents, each of which represents a therapeutic hypothesis that merits further exploration. Protein synthesis inhibitors and DNA inhibitors were among those found to mimic the transcriptional effects of *SETBP1* mutations. In contrast, compounds that disrupt DNA replication and transcription, including kinase inhibitors, topoisomerase inhibitors and RNA polymerase inhibitors, demonstrated strong opposing signatures, suggesting their potential as therapeutic candidates. These findings align with existing literature on the role of SETBP1 protein in transcriptional and epigenetic regulation^4^ as well as the inhibitory effect of *SETBP1* mutations on PP2A—a phosphatase responsible for dephosphorylation and inactivation of kinases. Given the enrichment of NF‐κB pathway genes in the shared *SETBP1* module, we examined NF‐kB inhibitors in Connectivity Map and observed that their effects are highly context‐dependent across cell lines (Figure S4). Nonetheless, several NF‐κB inhibitors exhibited relatively consistent opposing signatures to the shared *SETBP1* module and may warrant further investigation.

We were able to distil the transcriptional effects of leukaemogenic *SETBP1* from natural variation, combining high‐resolution quantitative molecular methods with rare patient samples. We overcame the barrier of accessing exceptionally rare samples through collaboration with the Schinzel–Giedion Syndrome Foundation, a charity founded by parents who have children with SGS. We observed that transcriptional changes associated with *SETBP1* hotspot activating mutations, in both germline and somatic contexts, converge on a shared inflammatory signature enriched for TNF‐alpha/NF‐kB signalling. This module is consistently enriched in samples where *SETBP1* acts as the dominant driver. Beyond providing a precious community resource, our analyses generate specific therapeutic hypotheses for counteracting the effects of *SETBP1* mutations. A limitation of our study is that we examined blood samples instead of bone marrow, which restricts investigation of early progenitor populations. Collecting bone marrow from healthy children is ethically challenging, however, especially in children with SGS where anaesthesia poses even greater risks. Future studies with larger, more systematically curated datasets will be needed to refine the transcriptional signature and its utility for prioritising therapeutic strategies. Given our focus on the direct transcriptional effects of *SETBP1* mutations, we did not examine potential changes in surrounding non‐mutant cells in the MDS sample. Overall, our work establishes a broader framework for systematically cataloguing the impact of cancer‐causing mutations from natural variation.

## AUTHOR CONTRIBUTIONS

S.B. and N.S. conceived and co‐directed the study. M.K.T. performed overall bioinformatic data analyses and interpreted the results and aided by S.B., N.D.A., M.D.Y. and H.J.W. C.P., T.O., A.O., D.Z., E.R.R., A.H., R.T., K.S., S.A., J.B. and N.S. collected and processed the samples for sequencing experiments. S.B. and M.K.T. wrote the manuscript. All authors have read and approved the manuscript.

## FUNDING INFORMATION

This study was funded by the Wellcome Trust (institutional grant to the Wellcome Sanger Institute (WT206194 and WT220540) and personal fellowship to S.B. (223135/Z/21/Z)). This research was supported by the NIHR GOSH Biomedical Research Centre and NIHR Cambridge Biomedical Research Centre (NIHR203312). The views expressed are those of the authors and not necessarily those of their funders.

## CONFLICT OF INTEREST STATEMENT

All authors declare that they have no competing interests.

## Supporting information


Appendix S1.

## Data Availability

Whole‐genome DNA sequencing (WGS) and single‐cell RNA sequencing (scRNA‐seq) data generated in this study have been deposited in the European Genome‐Phenome Archive (EGA) under accession numbers EGAD00001015829 (WGS) and EGAD00001015482 (scRNA‐seq). Single‐cell RNA‐seq data of healthy paediatric peripheral blood were obtained from Wang et al.^10^ via the Gene Expression Omnibus under accession number GSE168732. Single‐cell RNA‐seq and WGS data of myeloid neoplasms with and without *SETBP1* mutation were obtained from Trinh et al.^11^ via the EGA under accession numbers EGAD00001015453 (WGS) and EGAD00001015452 (scRNA‐seq). All code used to reproduce the analysis and figures described in this manuscript is available at https://github.com/miktrinh/SETBP1_GoF_mutations.
