# Supplementary material for: Single‐cell transcriptional consequences of leukaemogenic SETBP1 mutations
Source: Br J Haematol. 2026 Mar 25;208(6):2269–74. doi: 10.1111/bjh.70452 (PMC13267461; doi:10.1111/bjh.70452)
Supplement: Supplementary file 1 — Appendix S1. [file BJH-208-2269-s001.zip › bjh70452-sup-0001-TableS1-S3-FigureS1-S4@3_Supplementary_Materials_260211.docx]

**Single cell transcriptional consequences of leukaemogenic *SETBP1* mutations**

**Authors:**  Mi K. Trinh^1^, Nathaniel D. Anderson^1^, Matthew Young^1^, Holly J. Whitfield^1^, Conor Parks^1^, Toochi Ogbonnah^1^, Agnes Oszlanczi^1^, Di Zhou^1^, Emilia Robertson^2^, Angus Hodder^1,2^, Rebecca Thomas^2^, Karin Straathof^3,4^, Stuart Adams^2^, Jack Bartram^2,5^, Nuala Summerfield^6^, Sam Behjati^1,2,7,8^*

**Affiliations**:

^1^Wellcome Sanger Institute; Hinxton, CB10 1SA, UK.

^2^Great Ormond Street Hospital for Children NHS Foundation Trust, London, WC1N 3JH, UK.

^3^UCL Cancer Institute, 72 Huntley St, London, WC1E 6DD, UK.

^4^Great Ormond Street Biomedical Research Centre, 30 Guilford Street, London, WC1N 1EH, UK.

^5^UCL Great Ormond Street Institute of Child Health; London, WC1N 1EH, UK.

^6^The Schinzel-Giedion Syndrome Foundation, West Sussex, UK.

^7^Department of Paediatrics, University of Cambridge, Cambridge, CB2 0QQ, UK.

^8^Cambridge University Hospitals NHS Foundation Trust, Cambridge, CB2 0QQ, UK.

* Corresponding author. Contact addresses: sb31@sanger.ac.uk

# SUPPLEMENTARY TABLES

**Supplementary Table 1:** Summary of samples generated in this study and published datasets utilised.

**Supplementary Table 2**: List of markers associated with germline *SETBP1*-mutated peripheral blood cells compared to their normal counterparts.

**Supplementary Table 3**: List of significantly differentially expressed genes in bulk transcriptomic data of aCML samples with *SETBP1* hotspot mutations compared to those with wild-type *SETBP1*.

# SUPPLEMENTARY FIGURE LEGEND

**Supplementary Figure 1: SGS and MDS single-cell RNA-seq dataset**

1. Variant allele frequency (VAF, y-axis) of *SETBP1* mutations detected in samples from our cohort. The *SETBP1* mutations in samples from children with SGS exhibit VAF around 0.5, consistent with these being germline mutations. The *SETBP1* mutation in the MDS sample (PD61858) has a VAF = 0.18, indicating that the mutation is only present in approximately 36% of the cells, consistent with a somatic mutation. No *SETBP1* mutations were called in samples from the healthy donor and our other MDS case (PD61857), and therefore, these are not present in the plot.
2. Uniform Manifold Approximation and Projection (UMAP) visualisation (Harmony-integrated) of the SGS scRNA-seq dataset (detailed in **Table 1, Supplementary Table 1**), where cells (dots) are coloured by (top) distinct cell types and (bottom) donor ID.
3. Dot plot showing the z-scaled mean expression levels (colour) of key cell-type defining marker genes across cells from our SGS and MDS scRNA-seq datasets (as shown in **Figure 1b**). Dot size represents the proportion of cells within each category with positive expression.
4. Boxplots showing the cell type composition from each donor (individual dots), grouped by condition categories: SGS - germline *SETBP1* hotspot mutation; Atypical SGS - germline *SETBP1* non-hotspot mutation; Healthy - no *SETBP1* mutation. Asterisk (*) indicates samples from published scRNA-seq dataset of peripheral blood of healthy children (Wang et al., 2021) (1). Lineage proportions between samples with germline *SETBP1* mutation were compared against those from healthy individuals, using Mann-Whitney U test.


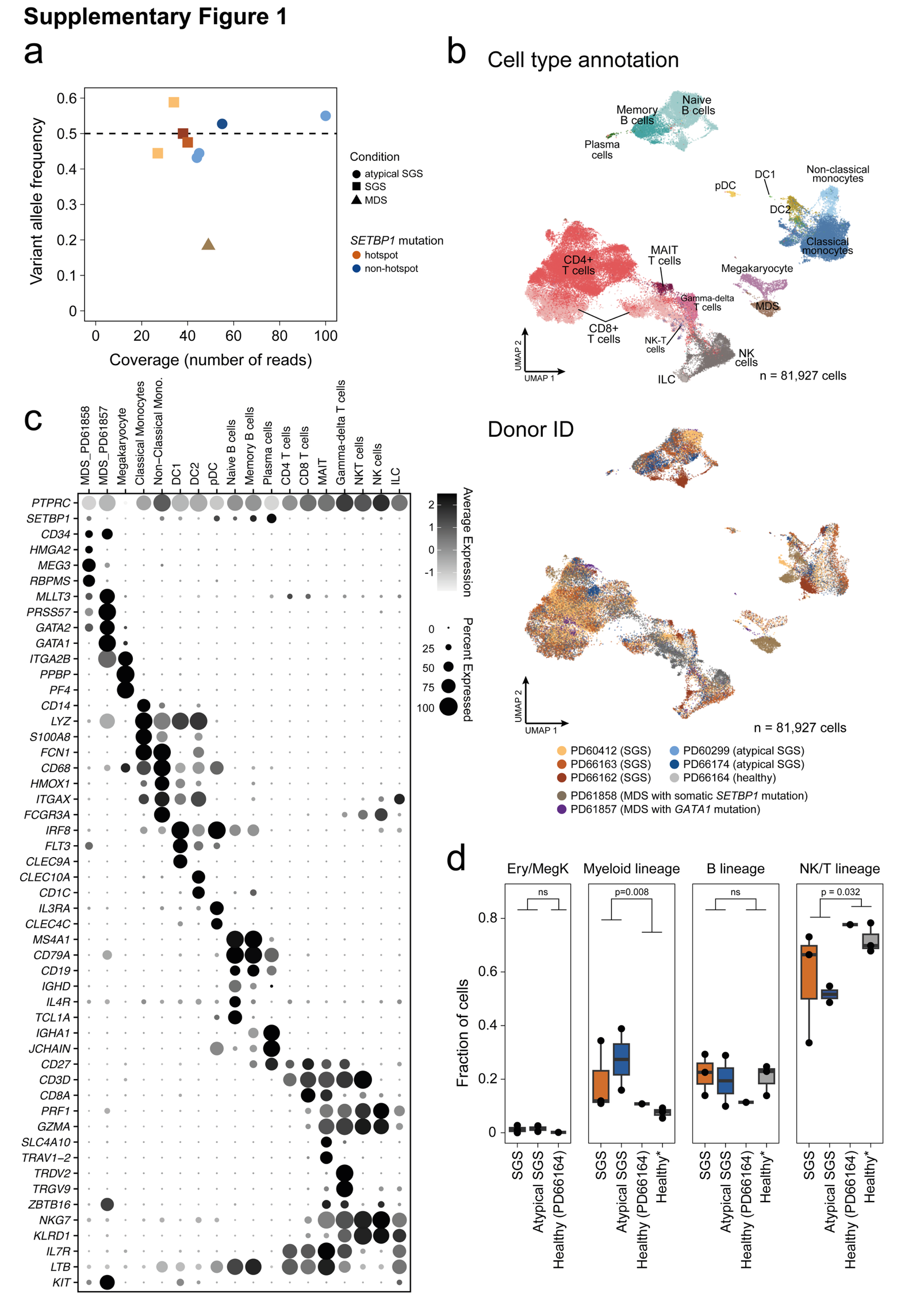


**Supplementary Figure 2: *SETBP1* expression level across cell types and MSigDB Hallmark enrichment in lymphoid germline module**

1. Boxplots showing the average normalised *SETBP1* expression (y-axis) across cells from each cell type per individual (dots). Samples are grouped on the x-axis by condition: MDS - two cases of MDS, with and without somatic *SETBP1* mutation; SGS - germline *SETBP1* hotspot mutation; Atypical SGS - germline *SETBP1* non-hotspot mutation; Healthy - no *SETBP1* mutation. Each dot represents one donor-cell-type combination; dot size indicates the fraction of cells from that cell type with positive *SETBP1* expression in that donor.
2. MsigDB Hallmark gene set enrichment analysis of up-regulated marker genes from the lymphoid germline *SETBP1* module (derived in **Figure 1c**). Only terms with significant enrichment (FDR < 0.1) are shown. Dot size corresponds to the number of up-regulated genes from the module present in each pathway, and colour indicates the fraction of genes in the pathway overlapped with up-regulated genes from the module. Pro-inflammatory signatures, including “TNF-alpha signalling via NF-kB”, are strongly enriched.

**
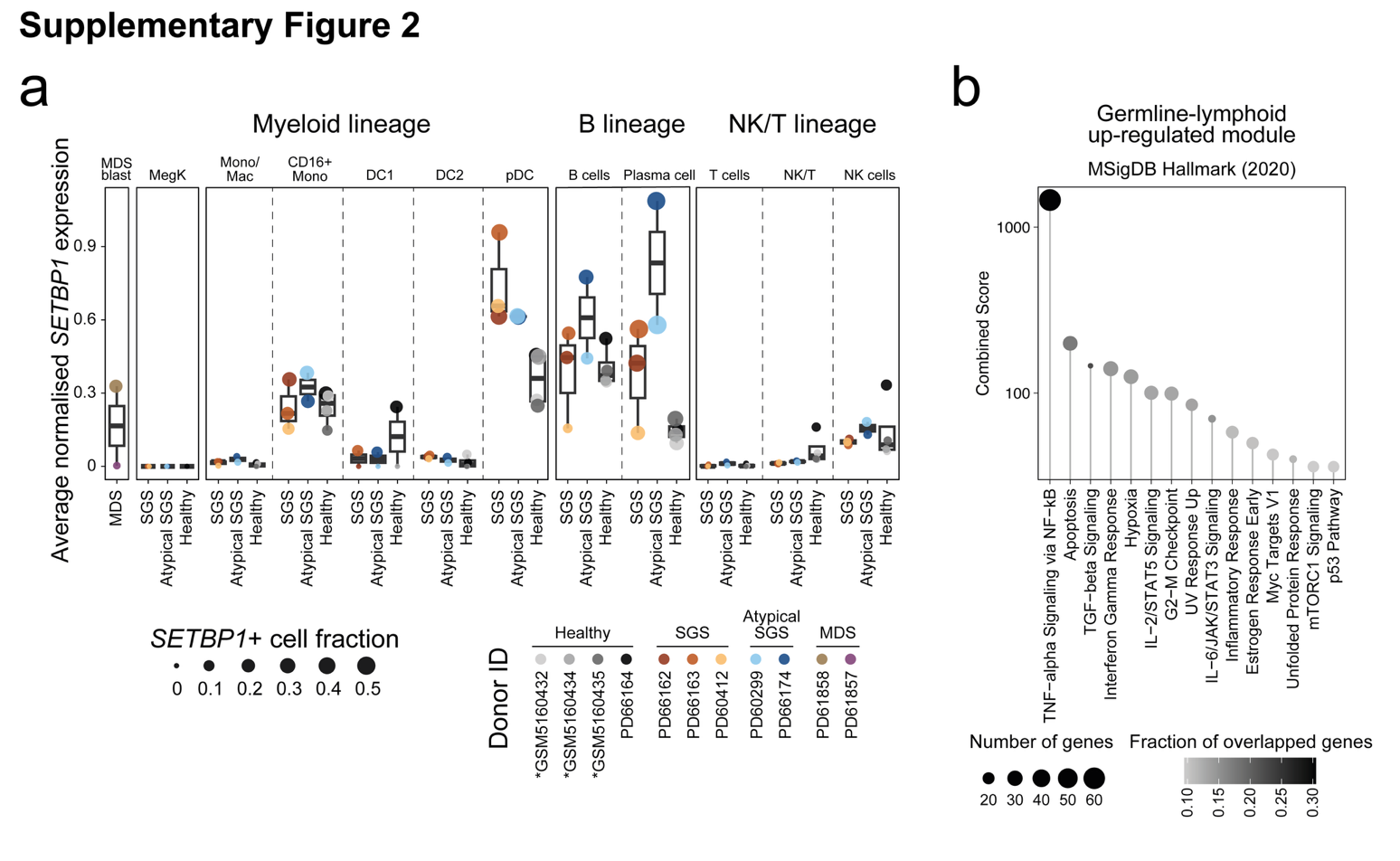
**

**Supplementary Figure 3: Enrichment of the shared *SETBP1* transcriptional modules in the BeatAML cohort.**

Enrichment scores (y-axis) of the somatic-germline shared myeloid *SETBP1* transcriptional module (as defined in **Figure 1e**) across samples from the BeatAML cohort, grouped by disease category (x-axis). Each point represents an individual sample, coloured by *SETBP1* mutation status: orange = hotspot mutation, blue = non-hotspot mutation, grey = wild-type. Boxes indicate the interquartile range (IQR) of enrichment scores within each group, the horizontal line within each box shows the median, and whiskers extend to the most extreme values within 1.5 × IQR.


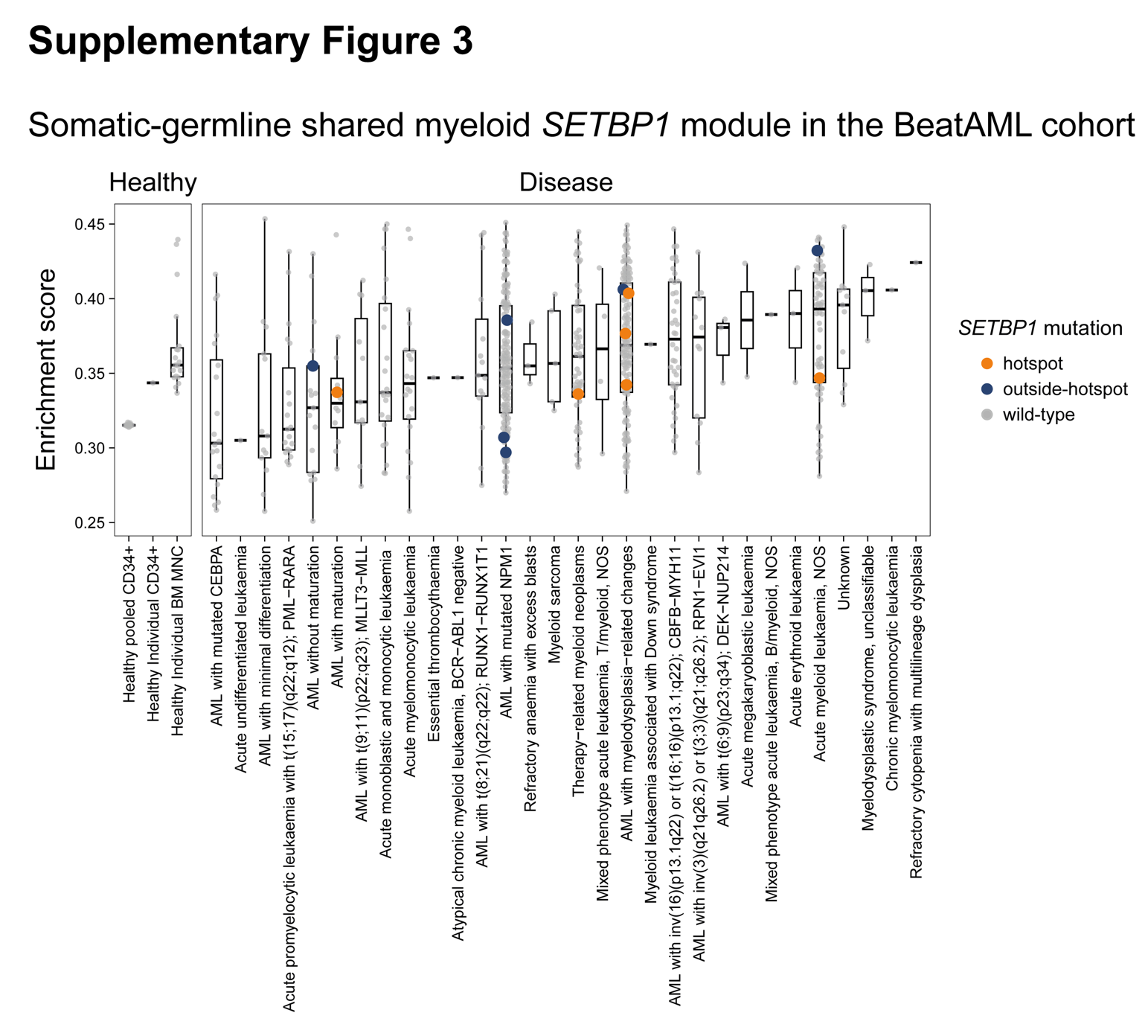


**Supplementary Figure 4: Connectivity Map perturbation signatures.**

Boxplots summarise normalised connectivity scores (x-axis) between the shared *SETBP1* transcriptional module (as defined in **Figure 1e**) and perturbation-induced transcriptional signatures from the Connectivity Map (CMap) L1000 database. Each black dot represents a single perturbation signature (drug + cell line), and the boxplots show the distribution of the connectivity scores for each compound. Compounds are shown if their absolute median normalised connectivity score > 1 (red and blue groups), or if they are NF-kB inhibitors. Positive scores indicate that the perturbation transcriptional signature mimic the *SETBP1* shared module, whereas negative scores indicate opposing effects. Although NFkB inhibitors have strong context- and cell line- dependent effects, several inhibitors consistently show strong negative scores across cell lines.


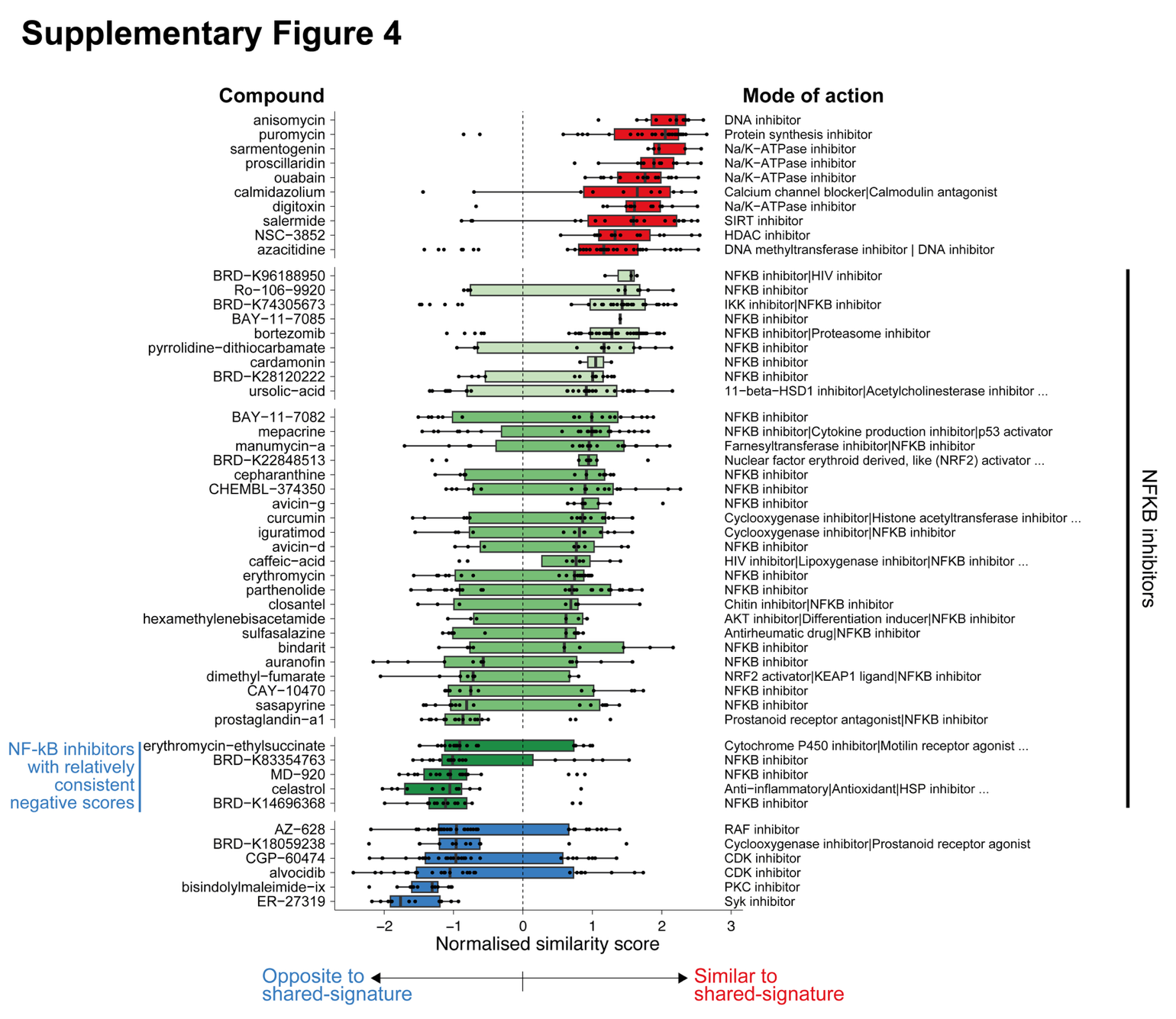


# METHODS

## **Sample acquisition, ethics and patient consent.**

Peripheral blood samples from children with SGS, atypical SGS, and one healthy donor were obtained from the COMBINEDBrain biorepository (<https://combinedbrain.org/biorepository>) through collaboration with The Schinzel-Giedion Syndrome Foundation (<https://sgsfoundation.org>), with appropriate written consent and approval from the London - Brent Research Ethics Committee (reference 16/LO/0960, source country Research Ethics Service reference MO: IORG0010151). Informed consent to participate in research was obtained from all patients or their legal guardians as stipulated by the study protocols.

## **10X single cell RNA sequencing (scRNA-seq)**

Peripheral blood mononuclear cells were prepared from peripheral blood samples by density centrifugation using Lymphoprep (Stemcell) according to manufacturer’s instructions, generating single-cell suspensions. The suspension was passed through a 70μm cell strainer (Falcon) and washed with PBS. If necessary, live cell enrichment using a Dead Cell Removal kit (Miltenyi Biotec) and red blood cell removal using the eBioscience 10X RBC Lysis Buffer (Multi-species) were performed as per manufacturer's instructions. All enriched live cells were washed and counted using a hemocytometer with trypan blue, single cell suspensions were adjusted to 1000 cells/ul accordingly. Cells were loaded onto the Chromium 10X controller as per the standard protocol of the Chromium Single Cell 5’ Reagent kits (v2 chemistry) in order to capture between 7000 cells/chip position. All the following steps were performed according to the standard manufacturer protocol. Post GEM-RT clean-up, cDNA amplification, and 5′ gene expression library construction were carried out according to the manufacturer’s instructions. The resulting libraries were sequenced on the Illumina Novaseq 6000 platform, aiming for an average of 300,000 reads per cell.

## **Quality control and preprocessing of scRNA-seq data**

Raw sequencing data was processed and mapped to the GRCh38 2020-A reference genome using Cell Ranger pipeline (2). The filtered count matrix, outputted by Cell Ranger, was then further QC’ed using Seurat (v4.0.1) (3) in R (v4.0.4). Cells with <300 genes, <500 UMIs, or mitochondrial fraction exceeding 30% were removed. Scrublet (v0.2.3) (4) was used to identify doublets. Cells were excluded if identified as doublets by Scrublet, or having a doublet score > 0.5. Ambient mRNA contamination was removed with SoupX (v1.6.1) (5). High resolution clusters (resolution=10) with >50% cells failing QC were also excluded.

The data were log normalised and scaled, and principal components were calculated using highly variable genes, following the standard Seurat workflow. Louvain clustering was performed (resolution = 1), and a uniform manifold approximation and projection (UMAP) calculated, using the top 75 principal components. To aid UMAP visualisation, all in-house scRNA-seq datasets were integrated using the Harmony algorithm, with donor identity as the integration variable. Harmony-corrected embeddings were used for neighbour graph construction, clustering, and UMAP visualisation. Pre- and post-Harmony embeddings were inspected to confirm that cells of the same type from different donors co-clustered appropriately. No additional batch correction was applied, to preserve biological variation across samples with different genetic backgrounds.

## **Cell type annotation**

We performed a semi-automated cell type annotation using a label transfer approach, using Celltypist (6) and its built-in logistic regression model (“Immune_All_Low”) which was trained on the cross-tissue immune reference scRNA-seq atlas (6). The model was used to calculate predicted similarity scores for individual cells in our query dataset against each reference class (i.e. different cell types in the reference dataset). Cells were assigned the label of the class with the highest positive similarity score. Annotation was further refined by manually assessing the expression of well-established cell-type specific marker genes (**Supplementary Figure 1c**) in high-resolution Louvain clusters.

To evaluate *SETBP1* expression level across different cell types, we used the *AverageExpression* function from the Seurat package to calculate the average *SETBP1* expression for each cell type per donor. The results are presented in **Supplementary Figure 1e.**

To determine whether *SETBP1* mutations affect the proportions of haematopoietic lineages, we obtained additional scRNA-seq data from three healthy paediatric peripheral blood samples from Wang et al., 2021 (1). To quantify lineage composition in peripheral blood samples from children with SGS or atypical SGS and healthy donors, we grouped cells by donor ID and their corresponding lineages as follows:

- Erythroid – Megakaryocyte lineage: erythrocytes and megakaryocytes.
- Myeloid lineage: DC1, DC2, pDC, monocytes, and macrophages.
- B lineage: B cells and plasma cells.
- NK/T lineage: natural killer cells, T cells, and natural killer/T cells.

For each donor, the proportion of each lineage was calculated as the number of cells within that lineage divided by the total number of haematopoietic cells from that donor. To compare each lineage proportion between individuals with germline *SETBP1* mutations and those without, we performed a one-tailed Mann-Whitney U test using the *wilcox.test* function in R.

## **Whole genome sequencing**

Whole genome sequencing (WGS) was performed on all samples, following the standard protocol as previously described (7). In brief, DNA was extracted using the AllPrep DNA/RNA/Protein Mini Kit (QIAGEN) following the standard protocol, and short insert (~500bp) genomic libraries were generated. Finally, 150 bp paired-end sequencing was conducted on the Illumina NovaSeq 6000 platform according to Illumina’s standard library generation protocols (with PCR). The average sequence coverage was at least 30X per sample (details in **Supplementary Table 1**).

Raw DNA sequencing data were aligned to the GRCh38 (Ensembl 103) reference genome using the Burrows-Wheeler algorithm (BWA-MEM) (8).

## **Germline and somatic variants calling**

Single-nucleotide variants were called using the CaVEMan algorithm (v1.18.2) (9) for each individual (one healthy donor, three children with SGS, two children with atypical SGS, and one *SETBP1*-mutant MDS), the genetic phylogenetic relation was reconstructed based on somatic single-nucleotide variants (SNVs, also referred to as substitutions). Short insertions/deletions in each case were called using Pindel algorithm (v3.10.0) (10). To recover both germline and somatic variants, the analysis was performed in an unmatched fashion, in which each WGS sample against an *in silico* normal human reference genome. In addition to the inbuilt QC filters, we further applied a series of stringent filters to remove low quality variants, following the detailed workflow outlined in Coorens et al., 2019 (11). Briefly, we only retained variants with a high median alignment score of supporting reads (ASMD >= 140), and required that fewer than half of the reads were clipped (CLPM=0), as well as not falling within 10 base pair of a deletion or insertion called by Pindel. We then recounted across both samples the variant allele frequency of all substitutions with a cut-off for base quality of 25 and read mapping quality of 30. Variants were also filtered out if they were called in a region of consistently low depth across both samples (excluding copy number segments). Next, to distinguish germline from somatic substitutions, we fitted a binomial distribution to the combined read counts across all samples per SNV site, and applied a one-sided exact binomial test to calculate the probability that the SNV is consistent with being a germline variant. We specifically focused on variants in the *SETBP1* gene, each of which was visually inspected using the genome browser Jbrowse (12) to exclude further sequencing or mapping artefacts. The exact *SETBP1* mutation detected in each case (if present), along with its corresponding variant allele frequency, is detailed in **Supplementary Table 1**.

## **Sample-level genotyping**

To ensure that sequencing data (both WGS and scRNA-seq) from the same individuals are correctly labelled, sample-level genotyping was performed using the *matchBAMs* function from the R package *alleleIntegrator* (13).

## **Derivation of transcriptional effects induced by *SETBP1* mutations**

*Germline SETBP1-mutation transcriptional effects*

The transcriptional changes associated with germline *SETBP1* mutations, referred to as the germline *SETBP1* module, were derived separately for the myeloid and lymphoid. For each cell type, we identified markers associated with cells from individuals with germline *SETBP1* mutations compared to their counterparts from the healthy donor, using the *FindMarkers* function from the Seurat package. Genes were identified as markers of *SETBP1*-mutated cells if they meet the following criteria:

(1) absolute log_2_ fold change (log2FC) >= 0.3

(2) adjusted p-value < 0.05

(3) differential expression pattern was consistent with an increase in the percentage of expressing cells in the up-regulated group and vice versa.

Genes identified as markers in B cells, plasma cells, T cells, NK cells, or NK/T cells form the lymphoid germline module, whereas those in monocytes, macrophages, and dendritic cells form the myeloid module. The full list of genes in each module is provided in **Supplementary Table 2**.

*Somatic SETBP1-mutation transcriptional effects*

We derived the somatic *SETBP1* module by analysing the bulk transcriptomes of aCML obtained from Piazza et al., 2013 (14), comparing samples with *SETBP1* hotspot mutations against those with wild-type *SETBP1*. Only genes with sufficient expression level as determined by the *filterByExpr* function from edgeR (v3.32.1) (15) (using default parameters), were included in differential expression analysis. A generalised linear model was fitted to the resulting bulk data using the *glmQLFit* function with the model formula: “Gene expression ~ Intercept + *SETBP1*_mutation_status”, where *SETBP1*_mutation_status is a factor with two levels (hotspot-mutation, wild-type). The *glmQLFTest* function was used to conduct genewise statistical tests on the coefficients of the *SETBP1*_mutation_status factor. Genes were considered to be significantly differentially expressed if (1) log2FC >= 0.5, and (2) false discovery rate (post Benjamani-Hochberg P-value correction) <0.05. The full list of differentially expressed genes is provided in **Supplementary Table 3.**

The “shared *SETBP1* mutation gene module” was defined as genes that were dysregulated in the same direction in both the somatic and the germline myeloid modules. This identified 69 genes that were up-regulated in both aCML samples with somatic *SETBP1* mutations and myeloid cells carrying germline *SETBP1* mutations; while no common down-regulated genes were detected. Genes exclusive to the somatic module comprise the somatic-specific module, and those unique to the germline myeloid module form the germline-specific module.

Gene set enrichment analysis was performed on the up-regulated genes from each module: (1) shared, (2) somatic-specific, and (3) germline-specific (myeloid). We used the function *enrichr* from the R package enrichR (v3.0) (16), and pathways from “MSigDB_Hallmark_2020” databases (17), obtained through enrichR (16). Gene sets with significant enrichment (FDR < 0.1) and at least 5% overlapping genes were shown in **Figure 1f**.

## **Transcriptional module enrichment analysis**

The enrichment score for each of the three *SETBP1*-mutation gene modules described above (composed of both up- and down-regulated genes) in single-cell RNAseq data was calculated using the function *AddModuleScore_UCell* from the R package UCell (18) (v1.3.1). For the bulk transcriptome dataset, the enrichment scores were computed using the function *simpleScore* from the R package singscore (19) (v1.10.0).

## **Connectivity map**

Following the strategy detailed in Custers et al., 2021 (20), the 69 genes from the shared *SETBP1* module were submitted to the CLUE online query tool (21). We focused exclusively on perturbagen type ‘compounds’ with known mode of action. These compounds were ranked by the median normalised connectivity score (norm_cs) across multiple cell lines in the database. The top 17 compounds with the strongest positive or negative association (absolute median normalised connectivity score > 1) with the shared *SETBP1* module were shown in **Figure 2c**.

# REFERENCES

1. Wang Z, Xie L, Ding G, Song S, Chen L, Li G, et al. Single-cell RNA sequencing of peripheral blood mononuclear cells from acute Kawasaki disease patients. Nat Commun. 2021 Sep 14;12(1):5444.

2. Zheng GXY, Terry JM, Belgrader P, Ryvkin P, Bent ZW, Wilson R, et al. Massively parallel digital transcriptional profiling of single cells. Nat Commun. 2017 Jan 16;8(1):14049.

3. Hao Y, Hao S, Andersen-Nissen E, Mauck WM, Zheng S, Butler A, et al. Integrated analysis of multimodal single-cell data. Cell. 2021 Jun 24;184(13):3573-3587.e29.

4. Wolock SL, Lopez R, Klein AM. Scrublet: Computational Identification of Cell Doublets in Single-Cell Transcriptomic Data. Cell Syst. 2019 Apr 24;8(4):281-291.e9.

5. Young MD, Behjati S. SoupX removes ambient RNA contamination from droplet-based single-cell RNA sequencing data. GigaScience. 2020 Nov 30;9(12):giaa151.

6. Domínguez Conde C, Xu C, Jarvis LB, Rainbow DB, Wells SB, Gomes T, et al. Cross-tissue immune cell analysis reveals tissue-specific features in humans. Science. 2022 May 13;376(6594):eabl5197.

7. Young MD, Mitchell TJ, Vieira Braga FA, Tran MGB, Stewart BJ, Ferdinand JR, et al. Single-cell transcriptomes from human kidneys reveal the cellular identity of renal tumors. Science. 2018 Aug 10;361(6402):594–9.

8. Li H, Durbin R. Fast and accurate short read alignment with Burrows–Wheeler transform. Bioinformatics. 2009 Jul 15;25(14):1754–60.

9. Jones D, Raine KM, Davies H, Tarpey PS, Butler AP, Teague JW, et al. cgpCaVEManWrapper: Simple Execution of CaVEMan in Order to Detect Somatic Single Nucleotide Variants in NGS Data. Curr Protoc Bioinforma. 2016 Dec 8;56:15.10.1-15.10.18.

10. Ye K, Guo L, Yang X, Lamijer EW, Raine K, Ning Z. Split-Read Indel and Structural Variant Calling Using PINDEL. In: Bickhart DM, editor. Copy Number Variants: Methods and Protocols [Internet]. New York, NY: Springer New York; 2018. p. 95–105. Available from: https://doi.org/10.1007/978-1-4939-8666-8_7

11. Coorens THH, Treger TD, Al-Saadi R, Moore L, Tran MGB, Mitchell TJ, et al. Embryonal precursors of Wilms tumor. Science. 2019 Dec 6;366(6470):1247–51.

12. Diesh C, Stevens GJ, Xie P, De Jesus Martinez T, Hershberg EA, Leung A, et al. JBrowse 2: a modular genome browser with views of synteny and structural variation. Genome Biol. 2023 Apr 17;24(1):74.

13. Trinh MK, Pacyna CN, Kildisiute G, Thevanesan C, Piapi A, Ambridge K, et al. Precise identification of cancer cells from allelic imbalances in single cell transcriptomes. Commun Biol. 2022 Sep 7;5(1):1–8.

14. Piazza R, Valletta S, Winkelmann N, Redaelli S, Spinelli R, Pirola A, et al. Recurrent SETBP1 mutations in atypical chronic myeloid leukemia. Nat Genet. 2013 Jan;45(1):18–24.

15. Robinson MD, McCarthy DJ, Smyth GK. edgeR: a Bioconductor package for differential expression analysis of digital gene expression data. Bioinformatics. 2010 Jan 1;26(1):139–40.

16. Chen EY, Tan CM, Kou Y, Duan Q, Wang Z, Meirelles GV, et al. Enrichr: interactive and collaborative HTML5 gene list enrichment analysis tool. BMC Bioinformatics. 2013 Apr 15;14:128.

17. Liberzon A, Birger C, Thorvaldsdóttir H, Ghandi M, Mesirov JP, Tamayo P. The Molecular Signatures Database Hallmark Gene Set Collection. Cell Syst. 2015 Dec 23;1(6):417–25.

18. Andreatta M, Carmona SJ. UCell: Robust and scalable single-cell gene signature scoring. Comput Struct Biotechnol J. 2021 Jan 1;19:3796–8.

19. Foroutan M, Bhuva DD, Lyu R, Horan K, Cursons J, Davis MJ. Single sample scoring of molecular phenotypes. BMC Bioinformatics. 2018 Nov 6;19(1):404.

20. Custers L, Khabirova E, Coorens THH, Oliver TRW, Calandrini C, Young MD, et al. Somatic mutations and single-cell transcriptomes reveal the root of malignant rhabdoid tumours. Nat Commun. 2021 Mar 3;12(1):1407.

21. Subramanian A, Narayan R, Corsello SM, Peck DD, Natoli TE, Lu X, et al. A Next Generation Connectivity Map: L1000 Platform and the First 1,000,000 Profiles. Cell. 2017 Nov 30;171(6):1437-1452.e17.
